# Study on the production of high 3HV content PHBV *via* an open fermentation with waste silkworm excrement as the carbon source by the haloarchaeon *Haloferax mediterranei*

**DOI:** 10.3389/fmicb.2022.981605

**Published:** 2022-08-17

**Authors:** Shuangfeng Cai, Yaran Wu, Runjie Liu, Hongzhe Jia, Yunxiao Qiu, Min Jiang, Yuwen Ma, Xingxu Yang, Siyu Zhang, Yan Zhao, Lei Cai

**Affiliations:** ^1^Engineering Research Center of Molecular Medicine of Ministry of Education, Key Laboratory of Fujian Molecular Medicine, Key Laboratory of Xiamen Marine and Gene Drugs, School of Medicine, Huaqiao University, Xiamen, China; ^2^School of Food Science and Biotechnology, Zhejiang Gongshang University, Hangzhou, China

**Keywords:** polyhydroxyalkanoate, haloarchaea, inexpensive carbon source, open fermentation, silkworm excrement

## Abstract

Silkworm excrement is hard to be degraded or bio-utilized by environmental microorganisms due to its high content of heavy metals and antimicrobial biomacromolecules in mulberry leaves. In traditional Chinese silk industry, the silkworm excrement results in environmental problems. In this study, the silkworm excrement after chlorophyll ethanol-extraction was researched. An open fermentation strategy was developed using the silkworm excrement as the sole or partial carbon source by haloarchaea to accumulate polyhydroxyalkanoates. As a haloarchaeon with strong carbon source utilization ability, *Haloferax mediterranei* was found to accumulate a certain amount of poly(3-hydroxybutyrate-*co*-3-hydroxyvalerate; PHBV) using waste silkworm excrement. The results showed that the addition of silkworm excrement into glucose based fermentation medium can significantly improve the production of PHBV. Using a mixture carbon source including the extract of silkworm excrement and glucose (with a 1:1 carbon content ratio), the yield of PHBV was 1.73 ± 0.12 g/l, which showed a 26% increase than that of fermentation without the silkworm excrement addition. When the NaCl content of medium was set to approximately 15%, fermentation without sterilization was performed using silkworm excrement as the carbon source. Moreover, the addition of the silkworm excrement extract could increase the 3-hydroxyvalerate (3 HV) content of PHBV regardless of the sterilization or non-sterilization fermentation conditions. When using silkworm excrement as the sole carbon source, the 3 HV content was as high as 16.37 ± 0.54 mol %. The real-time quantitative PCR results showed that the addition of the silkworm excrement could specifically enhance the expression of genes involved in the aspartate/2-ketobutyric acid pathway related to 3 HV synthesis in *H. mediterranei*, and further analysis of the amino acid of the silkworm excrement suggested that the high content of threonine in the silkworm excrement might be the reason for the increase of 3 HV content. Taken together, the success of non-sterile fermentation in hypersaline condition using haloarchaea implied a novel way to reuse the silkworm excrement, which not only reduces the production costs of PHBV, but also is conducive to environmental protection.

## Introduction

Mulberry leaves are the main feed of *Bombyx mori* (silkworm; Hu et al., 2021). Due to the characteristic of the digestive tract of silkworm, the silkworm excrement always contains large numbers of incompletely digested mulberry leaves, which are rich in nutrients (Park et al., 2011). However, the relatively high content of cadmium, arsenic, and other heavy metals are usually present in silkworm excrement because of the high heavy metals absorption capacity of the mulberry (Jiang et al., 2020), which makes silkworm excrement hard to be applied in biological reutilization. In the southwest of China, the massive stacking of silkworm excrement has brought serious environmental pollution (Hu et al., 2021). At present, the utilization of silkworm excrement for chlorophyll production has been well developed (Han et al., 2013b). However, there are few reports on how to reuse the excrement after chlorophyll extraction. In our previous study, two endogenous haloarchaea in silkworm excrement, *Haloarcula hispanica* A85 and *Natrinema altunense* A112, showed a polyhydroxyalkanoates (PHAs) accumulation ability when using waste silkworm excrement as the carbon source (Cai et al., 2021). However, due to the low PHAs yield of these two strains, the efficient conversion of silkworm excrement into PHAs by halophiles still needs further exploration.

PHAs are biodegradable polymers produced by many microorganisms as carbon storage under imbalanced nutrition conditions (Lee, 1996). PHAs have been widely applied in the production of degradable plastics. Compared with traditional petroleum-based plastics, PHA-based plastics have advantages for biocompatibility and good barrier properties (Cavalheiro et al., 2012). They can be extensively applied in biomedicine field (Mitra et al., 2021), such as cartilage repair and vascular scaffolds (Albuquerque and Malafaia, 2018). Therefore, PHAs are considered as environmentally friendly substitute for petroleum-based plastics (Benesova et al., 2017). Currently, there are still many difficulties in the PHAs production. The high cost is still a challenge for the large-scale commercial application of PHAs. Up to now, the cost of PHAs production is about 1.5–10.0 $/Kg_PHA_ (Hermann-Krauss et al., 2013; Bhattacharyya et al., 2015), which is much higher than that of traditional petroleum-based plastics (Policastro et al., 2021). The costs of raw materials and energy consumption of microbial fermentation usually take the most part of total costs. As the cost of carbon sources accounts for 30–40% of the total cost (Chanprateep, 2010; Policastro et al., 2021), the screening of inexpensive substrates seems to be an important direction to reduce costs, and many studies have tried to use cost-free waste carbon sources for PHA production, such as organic waste (Ganzeveld et al., 1999), wastewater from biodiesel industry (Martla et al., 2018) and digested food wastes (Du and Yu, 2002). Also, the use of waste silkworm excrement seems greatly reduce the production cost of PHAs (Cai et al., 2021).

So far, more than 150 monomer compositions have been found in PHAs, and the processability of PHAs varies with different monomer compositions. Among various kinds of PHAs, poly-3-hydroxybutyrate (PHB) and poly(3-hydroxybutyrate-*co*-3-hydroxyvalerate; PHBV) are the two most widely studied PHAs (Mitra et al., 2020). And PHBV was reported to possess better application potential than PHB, for example, PHBV with low 3 HV content (−5 mol %) can be used as bottle caps because of its hard texture, while PHBV with high 3 HV content (10 mol % – 20 mol %) can be used as bottle bodies due to its greater ductility (Barham et al., 1992). As a well studied PHBV-producing microorganism, haloarchaea has many advantages in the production of PHBV. First, haloarchaea can accumulate PHBV at the concentration of 10–25% sodium chloride (Zhao et al., 2013), which provides the possibility of open fermentation to produce PHBV. Secondly, the intracellular high salt environment makes the strain easy to be lysed in water, which simplified the PHA extraction (Hezayen et al., 2000; Han et al., 2007). Furthermore, compared with PHB-producing bacteria, haloarchaea can accumulate PHBV without adding 3 HV(3-hydroxyvalerate)-related carbon sources, such as valeric acid (Lu et al., 2008). Among them, *Haloferax mediterranei*, is one of the best-known PHBV producing haloarchaea. So far, the research on the production of PHBV in haloarchaea mainly focuses on the screening and modification of high-yield strains and the optimization of culture conditions. For instance, Zhao et al. constructed a strain with extracellular polysaccharide synthesis deficiency, *H. mediterranei* ∆*pyrF*ΔES1. Compared with the wild-type strain, the PHBV yield of this strain increased by 20% after optimizing the culture conditions (Zhao et al., 2013). However, not enough attention has been paid to saving the costs of carbon source in the production of PHBV by *H. mediterranei*. Thus far, only a few studies have attempted to produce PHBV with inexpensive carbon sources in *H. mediterranei* (Policastro et al., 2021). It was reported that olive mill wastewater can be converted into PHBV as the sole carbon source in shake flasks. After 96 h of fermentation, the maximum yield of PHBV is 0.2 g/l (Alsafadi and Al-Mashaqbeh, 2017). Wang et al. found that *H. mediterranei* can convert food waste into PHBV as a fermentation substrate. After 144 h of incubation, the PHBV yield is about 0.258 g/l and the 3 HV content is 10.7% (Wang and Zhang, 2020).

In the fermentation production of PHAs, the energy cost accounted for 8–11% of the total cost (Choi and Lee, 1997, 1999). While using waste raw materials as the carbon sources of fermentation, the energy cost rise to 25–30% (Naranjo et al., 2014). The requirement of non-sterile conditions in the reaction environment is crucial for the process scale-up. The high-salt environment in which haloarchaea grows provides the possibility for open fermentation. Therefore, using the high-salt growth conditions of halophiles and adopting open fermentation without sterilization is a way to reduce the energy cost of PHA production (Cai et al., 2021). And it was reported that *H. mediterranei* can use vinasse as a carbon source under non-sterile open fermentation conditions to obtain PHBV with 3 HV content of 12.36 mol% (Bhattacharyya et al., 2012).

PHBV has better ductility and lower melting point due to the insertion of the 3 HV monomer. Therefore, the control of 3 HV molar content has become a frontier research topic in the study of PHAs fermentation in haloarchaea (Han et al., 2015). Based on the well-developed genetic operating system, *H. mediterranei* has become a model strain to study the accumulation of PHBV. In recent years, the functional gene cloning of PHA synthase, the identification of PHA granule-binding proteins, and the study of 3HB (3-hydroxybutyrate) and 3 HV synthesis pathways have been accomplished by the use of nutrient-deficient strains and CRISPR-based systems (Hou et al., 2013; Liu et al., 2013a; Lin et al., 2021). It was found that there are four main pathways to synthesize propionyl coenzyme A (propionyl-CoA, the precursor of 3 HV) in *H. mediterranei*, including citramalate/2-oxobutyrate acid pathway (pathway I), aspartate/2-oxobutyrate pathway (pathway II), methylmalonyl-CoA pathway (pathway III) and 3-hydroxypropionate pathway (pathway IV; Han et al., 2013a; Hou et al., 2015). These studies provide theoretical basis and technical support for further exploring the changes of key pathways related to 3 HV synthesis in *H. mediterranei*.

In this study, we tried to use silkworm excrement as the main carbon source for a non-sterile fermentation of PHBV by *H. mediterranei* ATCC 33500. Combined with the analysis of PHBV synthesis pathway, the induction mechanism of the high 3 HV content PHBV accumulation by silkworm excrement addition was clarified at the gene transcription level. These can not only realize the reuse of waste, reduce the consumption of non-renewable resources, and greatly reduce the cost of carbon sources and energy, but also provide a novel research idea for the accumulation of the PHBV with adjustable 3 HV content to control its material properties.

## Materials and methods

### Pretreatment of silkworm excrement

All the silkworm excrement used in this study was the waste residue after the extraction of chlorophyll by ethanol method, which was provided by Fengming Chlorophyll Company Limited (Haining, Zhejiang Province, China). The extracts were obtained according to the method mentioned in the previous study (Cai et al., 2021). After shaking at 200 rpm for 2 h, the extracts were filtered through analytical filter paper to remove the insoluble residue, and were analyzed by the phenol-sulfuric acid method and Kjeldahl nitrogen determination method, respectively. The total sugar content of the water-soluble fraction of silkworm excrement (50 g/l) was quantified to be 9.45 ± 0.25 g/l, and the total nitrogen content was 0.87 ± 0.04 g/l.

### Strains and culture conditions

*H. mediterranei* was used for all subsequent experiments. The strain *H. mediterranei* ∆*pyrF*∆EC is a PHA synthase-deficient mutant strain of *H. mediterranei* ∆*pyrF* (Cai et al., 2012). The above archaea strains were obtained from Xiang’s Lab, Chinese Academy of Sciences (Beijing, China). The strains *H. hispanica* A85 and *N. altunense* A112 are PHA-accumulating haloarchaea. They were isolated from silkworm excrement previously, and stored in our lab (Cai et al., 2021).

All strains were first inoculated in nutrient-rich AS-165 medium (Cai et al., 2012; per liter, 150 g of NaCl, 20 g of MgSO_4_·7H_2_O, 2 g of KCl, 1.2 g of sodium glutamate, 5 mg of FeSO_4_·7H_2_O, 0.036 mg of MnCl_2_·4H_2_O, 3 g of trisodium citrate, 5 g of yeast extract, and 5 g of casamino, pH 7.0.) at 37°C and 200 rpm until the late logarithmic growth phase. Then strains were grown in PHA production medium (MGL medium; Cai et al., 2015), (per liter, 150 g of NaCl, 9.6 g of MgCl_2_, 14.4 g of MgSO_4_·7H_2_O, 5 g of KCl, 1 g of CaCl_2_, 3 g of yeast extract, 2 g of NH_4_Cl, 0.0375 g of KH_2_PO_4_, 10 g of glucose, 3 g of PIPES, 5 mg of FeSO_4_·7H_2_O, and 0.036 mg of MnCl_2_·4H_2_O, pH 7.0) and harvested to analyze for PHA accumulation after fermentation at 37°C for several days. All the fermentation experiments in this study were completed in 250 ml or 500 ml shake flasks.

In order to study the effect of silkworm excrement as microbial fermentation carbon source on the accumulation of PHA by strain ATCC 33500, three kinds of media were obtained using silkworm excrement and glucose as the sole or partial carbon source. The main carbon source in the MGL medium, 10 g/l glucose, is replaced by 53 g/l silkworm excrement to maintain the same carbon content, which is called SE medium. SM medium was set by mixing MGL medium with SE medium in equal volume. HMGL medium was generated by replacing 10 g/l glucose in MGL medium with 5 g/l.

### Detection of PHA using microscopy approach and gas chromatography

The nile red stained method was used for the preliminary detection of intracellular PHA accumulation (Salgaonkar et al., 2013). The strain ATCC 33500 was inoculated into AS-165 medium at 37°C for 48 h, then seed culture was inoculated into the MGL medium at a ratio of 1:10. After 48 h culture, the cells were collected by centrifugation at 13000 g, then resuspended by 15% NaCl solution, added with 20% Nile red solution (0.1 mg/ml), and kept in the dark for 20 min. Finally, stained cell samples were analyzed by fluorescence microscopy (Leica DM4; Wetzlar, Germany). In order to eliminate the false-positives caused by the non-specific binding of nile red to cell membrane, *H. mediterranei* ∆*pyrF*ΔEC was used as a negative control (Lu et al., 2008).

The accumulation of PHA was quantified by Agilent 7890A chromatograph (Agilent Technologies, Inc., Santa Clara, United States). The fermented broth was centrifuged at 12,000 × *g* for 15 min and the cells were harvested *via* centrifugation (12,000 × *g* for 15 min) and freeze-dried. The operation of esterification reaction and gas chromatography follows the previously reported protocol (Han et al., 2007; Juengert et al., 2018). Briefly, 80 mg lyophilized sample was transferred to a 10 ml esterification tube, 2 ml of chloroform and 2 ml of methanolic solution containing 3% (v/v) concentrated sulfuric acid and 0.1% (v/v) benzoic acid were added, and the reaction was conducted at 100°C for 4 h. After the reaction, 1 ml of distilled water was added, and shaken thoroughly. After stratification, the lower organic phase was analyzed by gas chromatography. The GC parameters were set as follows (Han et al., 2007; Juengert et al., 2018): the injection volume of 1 μl; the inlet temperature of 200°C, the detector temperature of 220°C; initial column temperature of 80°C, dwell for l.5 min, then ramp up to 140°C at a rate of 30°C/min. The PHBV standard (Sigma Aldrich Catalog No: 403121, 12 mol % PHV content) was used as the standard sample.

### Salinity screening and CFU assay in open fermentation

In order to explore the possibility of open fermentation under high salt stress, the growth of endogenous microorganisms in silkworm excrement in different salinity media was monitored. Five AS-165 media with different NaCl concentrations were established, and their NaCl contents were 1, 5, 10, 15, and 20%, respectively. The silkworm excrement sample was diluted to 10^−0^–10^−5^ in a gradient manner, and 10 μl of each dilution was spread on AS-165 medium agar plates with different salinities. All culture plates were incubated at 37°C for 168 h.

After the initial screening of the suitable salinity of the SE medium, the possibility of open fermentation of strain ATCC 33500 in unsterilized silkworm excrement medium with different salinities was further studied. In this study, the CFU assay was used to detect the proportion of target strain in silkworm excrement medium (Liu et al., 2013a; Cai et al., 2021). The fermented broth at the endpoint was diluted in a certain gradient, coated on AS-165 agar plates with different salinities, and incubated at 37°C for several days. More than 20 single colonies were randomly selected from each plate for 16S rDNA identification. The number of ATCC 33500 colonies was divided by the total number of selected colonies to calculate the proportion of target strains. Meanwhile, the production of PHA in different salinity medium was quantified by the GC method.

### RNA extraction and RT-qPCR analysis

To evaluate the effect of silkworm excrement on the expression of PHA synthesis related genes in strain ATCC 33500, qPCR experiments were performed. Total RNA extraction of the strain ATCC 33500 was isolated by TRIzol reagent method (Cai et al., 2012). RNA concentration was detected by Nanodrop 2000 spectrophotometer (Thermo Fisher Scientific Co., Ltd., Walsham, Massachusetts, United States). The first strand cDNA was synthesized according to the manufactory’s instructions (Vazyme, Nanjing, Jiangsu). All real-time qPCR was performed on StepOne Real-time qPCR instrument (Thermo Fisher Scientific Co., Ltd., Walsham, Massachusetts, USA) using a high-efficiency SYBR fluorescent quantitative PCR mix QPK-201 (Vazyme, Nanjing, Jiangsu), using 16S rDNA gene as internal reference. The PCR procedure was set as follows (Cai et al., 2014): initial denaturation at 95°C for 10 min, followed by 40 cycles of denaturation at 95°C for 30 s, annealing at 55°C for 30 s, and extension at 72°C for 30 s. The melting curve was generated by linear heating from 70 to 95°C for 25 min, to confirm the product specificity. The primers of target genes used are listed in Table 1. Each reaction was repeated three times. The relative expression difference was calculated using the 2^−ΔΔCt^ method.

**Table 1:** All the primers used in this study.

Primer name	5′-3′ Sequence
16S-F	CGTCCGCAAGGATGAAA
16S-R	CAGCGTCGTGGTAAGGT
cimA-F	CGCAAGGGTGTCGTTCA
cimA-R	AGCGTCGTGGCTGGTTC
mcmB-F	GTCATCTACTCGGGTCTCCA
mcmB-R	TCCGTCCATCACCTTCG
asd-F	ACGAAGCGGCAAAGTGG
asd-R	CGAGGGAAGCGACGAAA
mgl/metC-F	GGATGGGTGCGATAAACA
mgl/metC-R	CTCGTAGACCTGCGTGAAG
korA-F	CGTGCTCATTGCACTTACA
korA-R	AGCCCGACCATTCCTT
pdhA/oxdhA-F	CAGCAAGCGAGTCCA
pdhA/oxdhA-R	CGTGCCTTCTCCAGTC
porA-F	TGGCTTCTCGCTTATGTC
porA-R	TGCTCGGGCTTGGTT
pdhA1-F	AAGGGTAACAAGATGC
pdhA1-R	TCACCGAAGTAACAGAG
pdhA2-F	TGAGCAGCCAGTTCAGATAC
pdhA2-R	GGGCGAGTTTGATGTCC

### Determination of amino acids composition in silkworm excrement

The content of amino acids in silkworm excrement was determined according to previous reports (Yan et al., 2013). Every 100 mg of freeze-dried silkworm excrement sample was put into the hydrolysis tube. After adding 10 ml of 6 mol/l hydrochloric acid and 3 drops of phenol reagent, the hydrolysis tube was frozen for 5 min, evacuated, filled with high-purity nitrogen, and sealed at 110°C for 22 h. After the reaction, it was cooled to room temperature, filtered, and diluted to 50 ml with distilled water. A total of 1 ml of filtrate was dried under vacuum at 40°C, which was then dissolved by adding 1 ml of sodium citrate solution with pH value of 2.2, and injected into the automatic amino acid analyzer L-8900 (Hitachi, Japan, Tokyo) for determination. The instrument parameters were set as follows: the injection volume is 50 μl, the column temperature is 50°C, the ninhydrin flow rate is 0.4 ml/min, and the buffer flow rate is 0.35 ml/min.

### Sequence analysis and satistical analysis

The primers used in this study were designed by Primer Premier 5 software. Sequence homology was analyzed by the BLAST service.^1^ All data were analyzed by one-way ANOVA statistical method using GraphPad Prism 9. The results shown represent mean values ± standard deviations of three independent experiments. *p* < 0.05 was considered to be significantly different.

## Results

### The growth of strain ATCC 33500 in silkworm excrement medium

**Figure 1:**
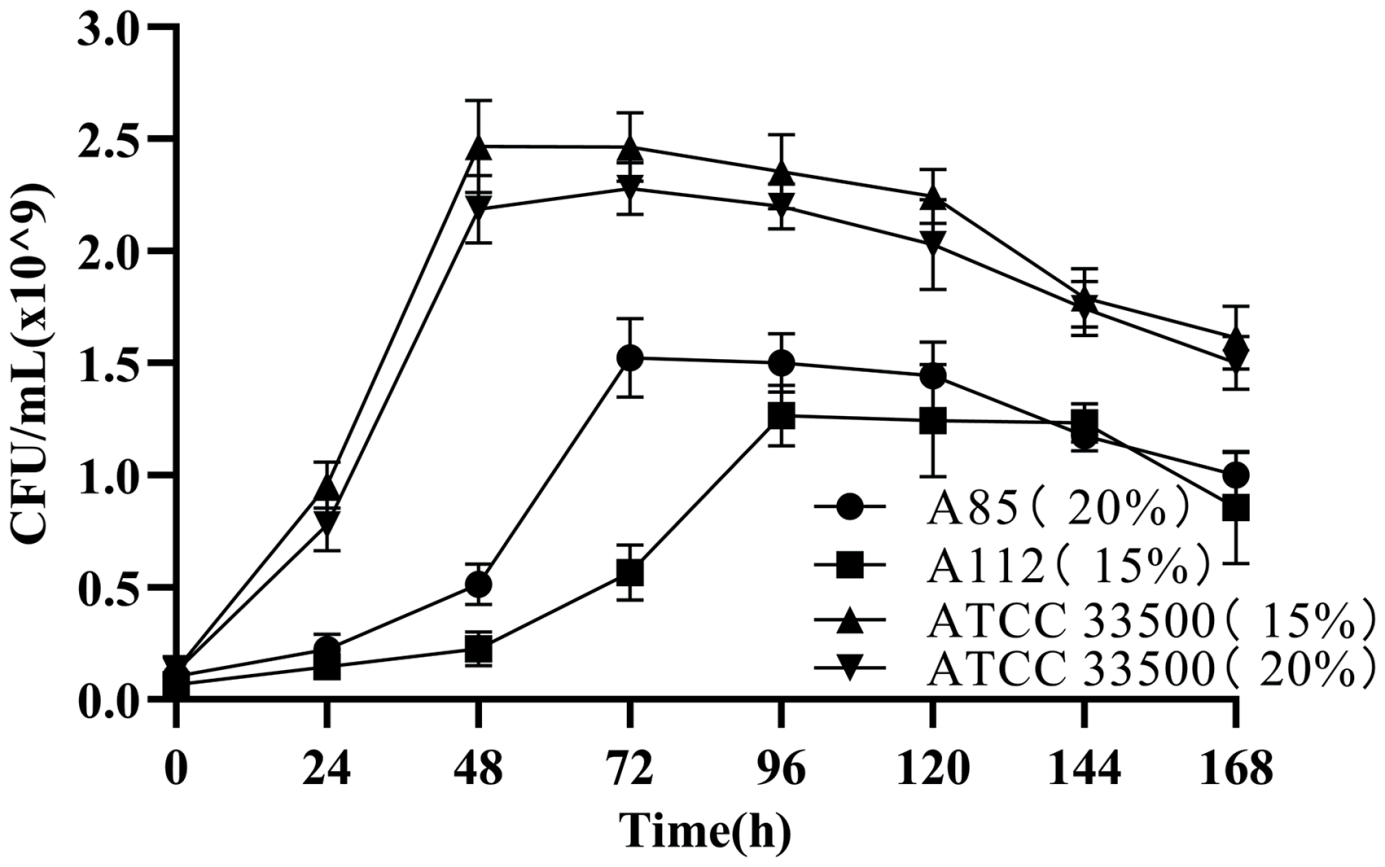
The growth of strains ATCC 33500(15% NaCl; 20% NaCl), A85(20% NaCl) and A112(15% NaCl) in SE medium were indicated by CFU counting. All the data are presented as means of duplicates with their standard errors.

The growth of strain ATCC 33500 in SE medium was investigated to determine whether the strain could use silkworm excrement as the sole carbon source. Two endogenous halophiles in silkworm excrement that are expected to accumulation PHB, *H. hispanica* A85 and *N. altunense* A112, were used as positive control in the medium with 15% or 20% NaCl content, respectively (Cai et al., 2021). The results showed that there seemed to be no significant difference in the growth of strain ATCC 33500 between 15 and 20% NaCl concentrations (Figure 1). The cell number of strain ATCC 33500 increased rapidly within 0–48 h of fermentation. After 48 h of incubation, the number of cells reached the maximum (about 2.4 × 10^9^ CFU/ml). After 96 h of incubation, the number of cells began to decrease slowly. In contrast, strain A85 grew slowly from 0 to 48 h at 20% NaCl concentration (Figure 1), and then grew rapidly after 48 h. The maximum cell number of the strain was 1.5 × 10^9^ CFU/ml when the incubation time reached 72 h. After 120 h of incubation, the number of cells began to decrease slowly with the extension of fermentation time. Strain A112, on the other hand, grew slowly in the early stage at concentration of 15% NaCl until 72 h, and then started to grow rapidly. After 96 h of fermentation, the maximum number of cells reached 1.25 × 10^9^ CFU/ml and maintained for several days (Figure 1). After 144 h of incubation, the number of cells of strain A112 decreased. The above results showed that strain ATCC 33500 could utilize silkworm excrement, and grew better in SE medium than strains A85 and A112, with shorter culture time and more cells.

### Study of PHA accumulation by strain ATCC 33500 using silkworm excrement as the sole carbon source

**Figure 2:**
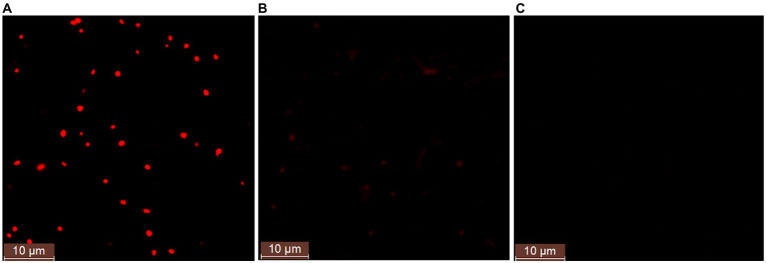
The fluorescence microscope examination of the Nile red stained cells in the fermented SE medium with the inoculation of strain *H. mediterranei* ATCC 33500 **(A)** or without **(B)**. The PHA-defeated strain *Haloferax mediterranei* ∆*pyrF*ΔEC **(C)** was used as a negative control to preliminarily determine the accumulation of PHA observed under fluorescence.

In order to further study whether strain ATCC 33500 could accumulate PHA using silkworm excrement as the sole carbon source, two methods were used for detection. Firstly, each fermention broth was stained with Nile Red solution and observed by fluorescence microscope. It was found that under the same microscope parameter setting, the fermentation broth inoculated with strain ATCC 33500 showed significant red fluorescence (Figure 2A), and the unsterilized SE medium without inoculation showed slight red dots (Figure 2B), while no red fluorescence was observed in the SE medium with inoculation of the PHA synthase-deficient mutant strain ∆*pyrF*ΔEC (Figure 2C). Meanwhile, the quantitative results of gas chromatography also revealed that strain ATCC 33500 could well utilize the silkworm excrement as the sole carbon source to accumulate PHA. The PHA production was 0.273 ± 0.02 g/l after 48 h of fermentation. After 72 h of culture, the PHA production was the highest, up to 0.368 ± 0.03 g/l. After 96 h of fermentation, the accumulation of PHA reduced slightly to 0.339 ± 0.02 g/l. However, without inoculation of seed medium, the production of PHA could not be detected in the fermented broth of the unsterilized SE medium (Table 2).

**Table 2:** Determination of the PHA accumulation by gas chromatography.

Strains	Hours	CDW (g/L)	PHA concentration (g/L)	PHA content (%)	3 HV Content (mol%)
ATCC 33500	48	7.40 ± 0.25	0.273 ± 0.02	3.70 ± 0.35	15.31 ± 0.31
	72	9.20 ± 0.47	0.368 ± 0.03	4.02 ± 0.51	16.24 ± 0.19
	96	8.44 ± 0.44	0.339 ± 0.02	4.02 ± 0.02	15.13 ± 0.34
ΔEC		n.d.	n.d.	n.d.	n.d.
CK		n.d.	n.d.	n.d.	n.d.

CDW, cell dry weight; CK, fermentation without seed culture inoculation; n.d., not detectable. All the data are presented as means of duplicates with their standard errors.

### Screen of suitable NaCl concentration of medium for open fermentation

**Figure 3:**
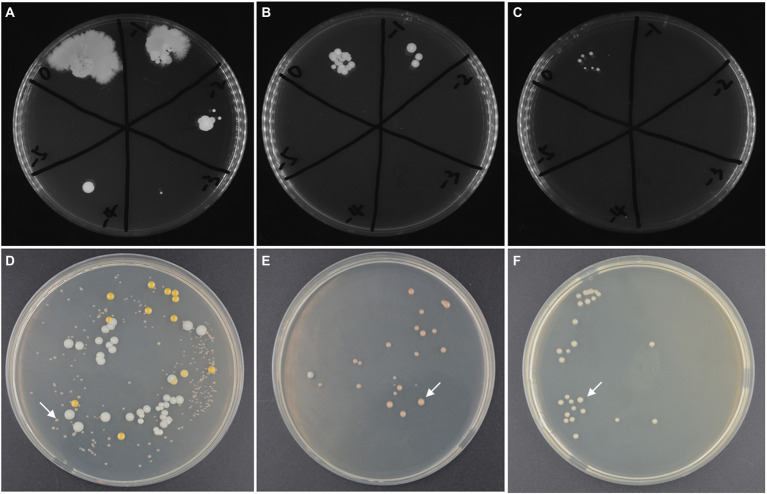
The microbial contamination in silkworm excrement medium with different salinity: **(A)** 10% NaCl; **(B)** 15% NaCl; **(C)** 20% NaCl and the microbial composition in open fermentation studied by CFU counting method after incubated for 168 h. **(D)** the fermented broths of the SE medium with 10% NaCl (72 h’ incubation); **(E)** the fermented broths of SE medium with 15% NaCl (120 h’ incubation); **(F)** the fermented broths of SE medium with 20% NaCl (120 h’ incubation). The target strain ATCC 33500 is indicated by white arrows **(D–F)**.

The high-salt environment in which haloarchaea grows provides the possibility for open fermentation. However, the higher NaCl concentration in the culture medium will also increases the costs of fermentation wastewater treatment. Therefore, it is necessary to screen the suitable concentration of NaCl. Firstly, without inoculating strain ATCC 33500, SE medium was fermented under open fermentation conditions for 72 h to obtain a certain range of NaCl concentration. The different settings for NaCl content were described previously. The results showed that the medium with NaCl concentration of 10, 15, and 20% could inhibit the growth of most endogenous and exogenous microorganisms in silkworm excrement medium. At the concentration of 10% NaCl, the number of cells was approximately 2 × 10^5^ CFU/ml, while the number of endogenous microorganisms detected at 15% or 20% NaCl concentration decreased significantly (Figures 3A–C). However, NaCl concentrations of 1 and 5% could not well inhibit the growth of endogenous microorganisms, and the number of cells reached more than 1 × 10^9^ CFU/ml (data not shown).

Further, in order to test whether the target strain takes ecological dominance at the end of open fermentation, the seed culture of strain ATCC 33500 was inoculated into unsterilized SE medium with different salinities. After 72 h of fermentation, the PHA yield and cell number ratio of strain ATCC 33500 were analyzed to evaluate the effect of open fermentation. The results of 72 h culture showed that when the NaCl concentration of SE medium was 10%, three dominant halophilic microorganisms were identified, including the target strain ATCC 33500, *Marinococcus halotolerans,* and *Alkalibacillus halophilus*, among which the target strain accounted for 75.44% ± 5.10% (Figure 3D). When the NaCl concentration of the SE medium was 15%, after 120 h of incubation, there were only the target strain ATCC 33500 and *Alkalibacillus halophilus*, and the proportion of strain ATCC 33500 was higher, 95.54% ± 1.21% (Figure 3E). After 120 h of incubation, all colonies identified in SE medium containing 20% NaCl were strain ATCC 33500 (Figure 3F).

GC analysis of the above three fermentation products with different salinities showed that the production of PHA in SE medium containing 10% NaCl was significantly decreased to 0.11 ± 0.01 g/l. However, when the NaCl concentration of SE medium was set to 15% or 20%, their PHA productions were similar, reaching 0.42 ± 0.02 g/l and 0.40 ± 0.04 g/l, respectively (Table 3). The above results showed that NaCl concentration below 10% could not inhibit the growth of endogenous microorganisms, and also affected the PHA accumulation of strain ATCC 33500. During open fermentation, there was no significant difference in the dominant proportion of target microorganisms or PHA yield between 15 and 20% NaCl concentration. Therefore, SE medium with low NaCl concentration (15%) was selected for the subsequent study.

**Table 3:** Determination of the PHA accumulation under different NaCl concentration.

Strains	NaCl concentration (%)	CDW (g/L)	PHA concentration (g/L)	PHA content (%)	3 HV content (mol%)
ATCC 33500	10	9.61 ± 0.14	0.11 ± 0.01	1.13 ± 0.08	5.44 ± 0.10
	15	11.17 ± 0.50	0.42 ± 0.02	3.80 ± 0.05	15.90 ± 0.33
	20	10.41 ± 1.26	0.40 ± 0.04	3.83 ± 0.12	15.49 ± 0.01
CK		n.d.	n.d.	n.d.	n.d.

CDW, cell dry weight; CK, fermentation without seed culture inoculation; n.d., not detectable. All the data are presented as means of duplicates with their standard errors.

### The PHA accumulation *via* open fermentation using silkworm excrement as the sole or partial carbon source by strain ATCC 33500

**Figure 4:**
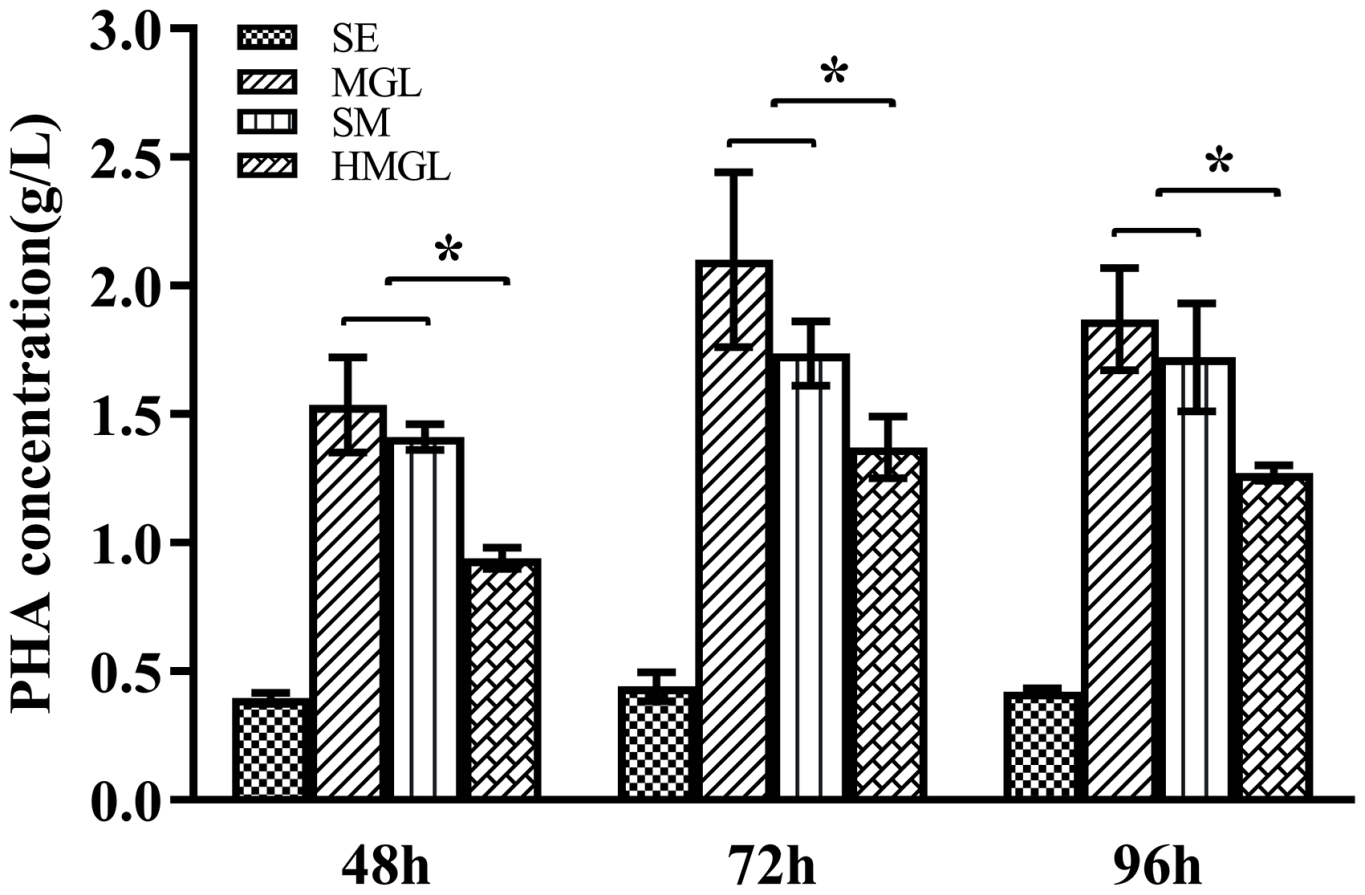
The PHA accumulation of strain ATCC 33500 in open fermentation using silkworm excrement as the carbon source. The PHA accumulation was detected by gas chromatography analysis in SE, MGL, SM, and HMGL medium. All the data are presented as means of duplicates with their standard errors [*significant differences (*p* < 0.05)].

To evaluate the effect of replacing classical carbon source glucose with silkworm excrement, SM, MGL, and HMGL media were introduced for further study. Open fermentation was performed in different media for 96 h. The results of the gas chromatography showed that the PHA yield of strain ATCC 33500 could reach 0.40 ± 0.01 g/l after fermentation on SE medium with silkworm excrement as the sole carbon source for 48 h (Figure 4). Meanwhile, the production of PHA in MGL, SM, and HMGL media was 1.53 ± 0.18 g/l, 1.41 ± 0.05 g/l, and 0.94 ± 0.04 g/l, respectively. After 72 h of incubation, the PHA production of strain ATCC 33500 was the highest in SE, MGL, SM and HMGL media, which were 0.44 ± 0.04 g/l, 2.10 ± 0.34 g/l, 1.73 ± 0.12 g/l, and 1.37 ± 0.12 g/l, respectively. Among them, the PHA yield in SM medium was approximately 26% higher than that in the fermentation without silkworm excrement. Additionally, it was also found that the PHA yield of the SM medium (1.72 ± 0.21 g/l) was similar to that of the MGL medium (1.87 ± 0.20 g/l) fermented for 96 h, and its PHA production was also higher than that of SE (0.42 ± 0.01 g/l) or HMGL (1.27 ± 0.03 g/l) medium (Figure 4).

As shown in Table 4, strain ATCC 33500 can not only use silkworm excrement to accumulate high-yield PHBV, but also has a higher 3 HV content in the accumulated PHBV. After 48–96 h of incubation, the highest 3 HV content accumulated in MGL medium was 10.90 ± 0.89 mol%, while that it could be as high as 16.37 ± 0.54 mol% in SE medium. The 3 HV content in HMGL medium was 8.33 ± 0.07 mol%, while when adding equal volume of silkworm excrement (53 g/l), the 3 HV content increased by 44.66% to 12.05 ± 0.09 mol%.

**Table 4:** The PHA accumulation in open fermentation.

	Hours	48		72		96	
Strains	Medium	PHA concentration (g/L)	3 HV content (mol%)	PHA concentration (g/L)	3 HV content (mol%)	PHA concentration (g/L)	3 HV content (mol%)
ATCC 33500	SE	0.40 ± 0.01	15.20 ± 0.07	0.44 ± 0.04	16.37 ± 0.54	0.42 ± 0.01	15.29 ± 0.03
	MGL	1.53 ± 0.18	10.34 ± 0.56	2.10 ± 0.34	10.90 ± 0.89	1.87 ± 0.20	10.12 ± 0.19
	SM	1.41 ± 0.05	12.05 ± 0.09	1.73 ± 0.12	11.68 ± 0.49	1.72 ± 0.21	11.67 ± 0.04
	HMGL	0.94 ± 0.04	8.33 ± 0.07	1.37 ± 0.12	8.05 ± 0.04	1.27 ± 0.03	7.98 ± 0.11
CK		n.d.	n.d.	n.d.	n.d.	n.d.	n.d.

CDW, cell dry weight; CK, fermentation without seed culture inoculation; n.d., not detectable. All the data are presented as means of duplicates with their standard errors.

### Effect of silkworm excrement on the increase of 3 HV content in PHBV of strain ATCC 33500

**Figure 5:**
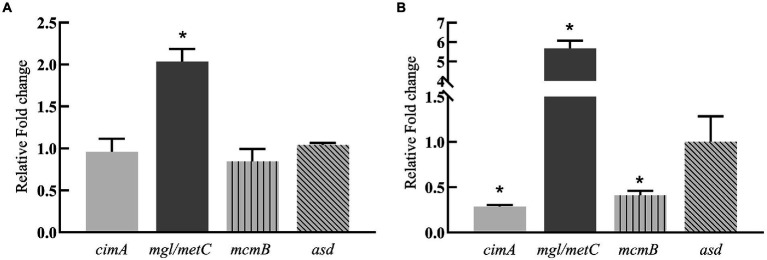
Relative expression levels of key genes (*cimA*; *mgl*/*metC*; *mcmB*; *asd*) in four pathways related to 3 HV synthesis in strain ATCC 33500 exposed to short-term **(A)** and long-term **(B)** silkworm excrement. All the data are presented as means of duplicates with their standard errors. [*significant differences (*p* < 0.05)].

To explore the reason for the increase of 3 HV content induced by silkworm excrement, genes related to the synthesis of 3 HV in strain ATCC 33500 were investigated. Firstly, in all four characterized 3 HV biosynthesis pathways (Han et al., 2013a), one key gene was selected for each pathway to detect changes in gene expression (pathway I: *cimA*; pathway II: *mgl/metC*; pathway III: *mcmB*; pathway IV: *asd*). After short-term or long-term induction with silkworm excrement extract, it was found that the expression of *mgl/metC* in pathway II was significantly up-regulated (Figure 5), with an increase of approximately 2–5 folds. In the short-term exposure stimulation experiment, the expression of *cimA* in pathway I and *mcmB* in pathway III decreased slightly, but in the long-term stimulation experiment, their expression was significantly down-regulated. Whether in short-term or long-term stimulation, the expression of *asd* in pathway IV remained unchanged (Figure 5).

**Figure 6:**
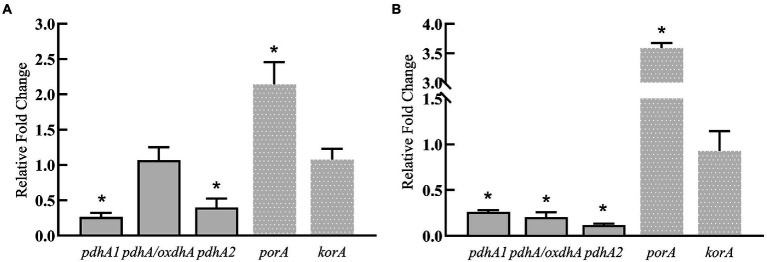
The relative expression levels of key genes (*pdhA*/*oxdhA*, *pdhA1*, *pdhA2*, *korA*, *porA*) in two pathways related to 3 HV synthesis in strain ATCC 33500 under short-term **(A)** and long-term **(B)** exposure to the silkworm excrement. All the data are presented as means of duplicates with their standard errors [*significant differences (*p* < 0.05)].

The above results show that silkworm excrement can enhancing the gene expression of aspartic acid/2-ketobutyric acid pathway, which indicates that silkworm excrement may increase the content of 2-ketobutyric acid. Then, five genes involved in the production pathway of propionyl-CoA from 2-ketobutyrate were further analyzed. It was shown that the expression of *porA* was significantly up-regulated after short-term or long-term induction by silkworm excrement, resulting in a 2.5–3.5 folds increase (Figure 6). The expression of *pdhA*/*oxdhA* did not change significantly after the short-term induction by silkworm excrement, but their expression was significantly down-regulated in the long-term induction experiment. In short-term or long-term silkworm excrement induction, it was found that the expression of *pdhA1* and *pdhA2* was significantly down-regulated, while the expression of *korA* did not change significantly (Figure 6).

**Figure 7:**
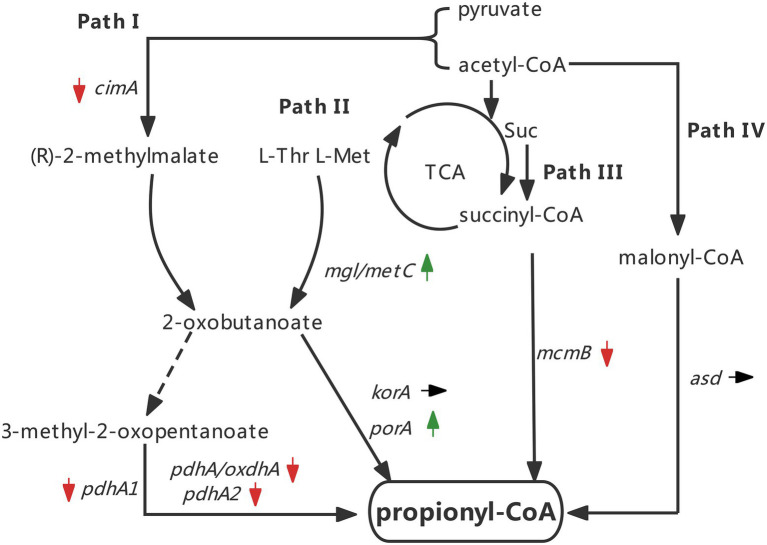
Changes of key genes expression in four pathways under the silkworm excrement exposure (Green arrow indicates up-regulation; red arrow indicates down-regulation; black arrow indicates no change).

Taken together, the expression changes of all tested genes are indicated by arrows (Figure 7). The results implied that silkworm excrement mainly stimulated the aspartate/2-ketobutyric acid pathway (pathway II) for the generation of 3 HV precursor propionyl-CoA in strain ATCC 33500, which may alter the content of 2-ketobutyric acid, and the 2-ketobutyric acid can be further decarboxylated by α-ketoglutarate/pyruvate:ferredoxin oxidoreductase to produce propionyl-CoA, thereby increasing the 3 HV content in PHBV (Figure 7).

### Determination of amino acid components in silkworm excrement

The analysis of pathway II of propionyl-CoA metabolic network suggests that exogenous amino acids in the medium may be the effector of the aforementioned 3 HV content enhancement (Figure 7). Amino acids such as threonine and methionine can synthesize propionyl-CoA *via* the aspartate/2-ketobutyric acid (pathway II), thereby increasing the 3 HV content of PHBV (Han et al., 2013a). The amino acid composition of silkworm excrement was analyzed by automatic amino acid analyzer (Biochrom Ltd., Cambridge, England). It was found that silkworm excrement mainly contained five amino acid components, among which valine (199.67 ± 1.04 μg) and threonine (199.22 ± 1.74 μg) showed the highest content (Table 5). This result was consistent with the predicted enhancement of propionyl-CoA pathway.

**Table 5:** Determination of the content of main amino acids in silkworm excrement.

Amino acid	Concentration μg/g
valine	199.67 ± 1.04
threonine	199.22 ± 1.74
glutamic acid	159.74 ± 2.06
aspartic acid	83.58 ± 1.30
methionine	58.61 ± 1.43

## Discussion

Due to the digestive characteristics of the silkworm, silkworm excrement usually contains abundant polysaccharides and amino acids (Park et al., 2011). So far, the main reutilization of silkworm excrement is ethanol extraction of chlorophyll (Han et al., 2013b). Since the silkworm excrement contains relatively high content of heavy metals, the silkworm excrement after chlorophyll extraction has become an industrial waste that is difficult to be reused biologically (Jiang et al., 2020). This study explored the possibility of using inexpensive silkworm excrement carbon sources and open fermentation strategy to produce PHA by halophiles to reduce fermentation cost. It was showed that waste silkworm excrement could be used to accumulate PHBV with high 3 HV content by haloarchaea, and the high salt fermentation medium of haloarchaea could ensure the possibility of open fermentation, thus reducing the energy consumption in the sterilization processes.

It was reported that some halotolerant bacteria, including *Halomonas janggokensis*, *Halomonas salina* and *Halomonas venusta* strains, have good PHB accumulation ability and salt tolerance characteristics (Tan et al., 2011; Martínez-Gutiérrez et al., 2018; Cai et al., 2021), but relevant studies showed that none of these bacteria could make good use of waste silkworm excrement to accumulate PHA (Cai et al., 2021). Two haloarchaea strains isolated from silkworm excrement, *H. hispanica* A85 and *N. altunense* A112, were considered to be able to use silkworm excrement as the sole carbon source for the growth and accumulation of PHB (Cai et al., 2021), but the yield was not enough for industrial production. After 96 h of fermentation, strain A85 could accumulate PHB of 0.31 ± 0.01 g/l in silkworm excrement medium with the concentration of 20% NaCl, while strain A112 had the highest PHB yield of 0.12 ± 0.02 g/l after 120 h of fermentation at the concentration of 15% NaCl. In this study, the model haloarchaea *H. mediterranei* can successfully utilize silkworm excrement as the sole carbon source to accumulate PHBV. After fermentation on the sterilized medium containing 15% NaCl for about 72 h, the PHBV yield of strain ATCC 33500 could reach 0.37 ± 0.03 g/l, which was significantly higher than that of endogenous microorganism isolated from silkworm excrement (Table 2). Compared with strains A85 and A112, strain ATCC 33500 could achieve higher fermentation yield in a shorter time. It was indicated that strain ATCC 33500 might have stronger ability to utilize complex carbon sources. On the other hand, it also meant that a lot of research is needed to further optimize the fermentation of strains A85 or A112. It is worth noting that when silkworm excrement was present in the culture medium, a significantly higher 3 HV content was detected (Table 2), whereas 3 HV monomers can hardly be detected using strains A85 and A112 under the same fermentation conditions (Cai et al., 2021), which may be due to the stronger ability of strain ATCC 33500 to synthesize propionyl-CoA through unrelated carbon sources (Hou et al., 2013). This is the first report on the conversion of agricultural waste silkworm excrement as the sole carbon source into PHBV by microbial fermentation.

The high salt environment of haloarchaea provides the possibility of open fermentation. In the silkworm excrement medium with the concentrations of 1% or 5% NaCl, the number of cells of endogenous microorganisms in silkworm excrement reached a very high level after 72 h of fermentation without inoculating any strains, which indicated that the open fermentation could not be completed well with the commonly reported halotolerant PHA accumulating bacteria. When the salinity increased to 10%, the number of endogenous microorganisms in the fermented silkworm excrement medium was approximately 2 × 10^5^ CFU/ml (Figure 3A). After inoculation with strain ATCC 33500, the number of cells of the target strain accounted for about 75% of the total (Figure 3D). Previous studies have shown that the number of cells of target strain A85 and A112 account for only about 60–70% in silkworm excrement medium containing 15 and 20% NaCl, respectively (Cai et al., 2021). In silkworm excrement medium with 15 and 20% NaCl concentration, the proportion of cell number of strain ATCC 33500 could reach more than 90–100% after 72 h of fermentation (Figures 3E,F). It seems that strain ATCC 33500 can successfully complete open fermentation at lower salinity and has better ecological dominance, which may be related to the ability of strain ATCC 33500 to secrete a halocin, named H4, in the late stage of fermentation (Cheung et al., 1997; Liu et al., 2013b). In previous studies, endogenous haloarchaea A85 and A112 of excrement medium were reported to be the dominant strains for high-salt fermentation in silkworm excrement medium (Cai et al., 2021). After the addition of strain ATCC 33500, it can be seen that the above two strains no longer exist in silkworm excrement medium, which also implied the possibility of strain ATCC 33500 secreting antimicrobial peptides. On the other hand, our study also found that strain ATCC 33500 had the strongest PHBV accumulation capability (0.42 ± 0.02 g/l) in the medium containing 15% NaCl, which was 4-fold higher than that in the medium containing 10% NaCl, and was slightly higher than that in the medium containing 20% NaCl (Table 3).

Finally, the open fermentation based on the optimal salinity showed that the PHA yield of strain ATCC 33500 in SE medium containing 15% NaCl for 48 h and 72 h was similar and up to 0.44 g/l. When an equal volume of silkworm excrement (53 g/l) was added to HMGL medium, the PHA yield in SM medium could reach the similar yield to that in MGL medium after 96 h of fermentation, which was 1.72 ± 0.21 g/l, which could reach 92% of the PHA yield in MGL medium and was also higher than that in SE or HMGL medium (Figure 4). The addition of cost-free silkworm excrement seems to largely save the amount of glucose added, which indicates that silkworm excrement contains enough carbon sources, that can be well utilized by the haloarchaeon *H. mediterranei* ATCC 33500. Some studies on PHB production with open fermentation have been reported in halophilic bacteria. *Halomonas campaniensis* LS21 can accumulate 2.77 g/l PHB by utilizing kitchen waste for open fermentation under high salt conditions (Yue et al., 2014). *Halomonas* sp. KM-1 had been proved to be effectively accumulate PHB by utilizing waste biodiesel glycerol, and the yields under non-sterilized condition is as high as 63.6% of the cell dry weight (Kawata and Aiba, 2010). *Halomonas* TD01 could accumulate PHB up to 80% of CDW during non-sterile fermentation with glucose supplementation (Tan et al., 2011). Although compared with bacteria, the yield in this study needs to be further improved, haloarchaea has obvious advantages in utilizing silkworm excrement or other waste to accumulate PHBV.

In this study, the 3 HV content of PHBV was 10.90 ± 0.89 mol% after 72 h of fermentation in MGL medium (Table 4), which was consistent with the relevant research reports (Han et al., 2015). In SE medium, the 3 HV content in PHBV reached 16.37 ± 0.54 mol% (Table 4), which was significantly higher than that in MGL medium. It was also slightly higher than the highest 3 HV content reported so far when using waste raw materials as the sole carbon sources to produce PHBV. As previously reported, the 3 HV content of PHBV in *H. mediterranei* was 15.4 mol% by using ethanol industrial waste stream as fermentation carbon source (Bhattacharyya et al., 2014). Furthermore, for *H. mediterranei*, it has been also reported that olive oil industrial wastewater could be used as the sole carbon source for the accumulation of PHA by one-stage culture, but the 3 HV content was only 6.5 mol% (Alsafadi and Al-Mashaqbeh, 2017).

When half amount of glucose was replaced by silkworm excrement (53 g/l), the 3 HV content of fermentation cells increased by approximately 45% (Table 4). The results showed that the extract of silkworm excrement may promote the synthesis of propionyl-CoA during the fermentation of strain ATCC 33500. By analyzing the propionyl-CoA biosynthesis pathways of strain ATCC 33500, the related key genes in all four classical pathways were selected to investigate the expression level of silkworm excrement extract induced cells. The results showed that *mgl/metC* and *porA* constituted an up-regulated pathway. In addition, there was no sign of enhancement in all other pathways, some of which were shown to be down-regulated (Figure 7). Further determination of amino acid components in silkworm excrement showed that silkworm excrement contained high contents of threonine, methionine, and other amino acids (Table 5), which may be a direct factor to promote the accumulation of PHBV with high 3 HV content in strain ATCC 33500. It has been reported that some bacteria could synthesize PHBV from unrelated carbon sources by regulating threonine synthesis and other related pathways. For example, the genetically engineered strain *halomonas* TD08 could increase the metabolic flux of propionyl-CoA by overexpressing the threonine synthesis pathway. Therefore, strain TD08 could use various carbohydrates as the sole carbon source to accumulate PHBV with 4–6 mol % 3 HV content (Tan et al., 2014). It was reported in archaea that threonine and methionine could be converted to 2-ketobutyric acid by threonine dehydratase or methionine-γ-cleavage enzyme, and then decarboxylated by α-ketoglutarate/pyruvate:ferredoxin oxidoreductase to eventually form propionyl-CoA (Han et al., 2013a). The genes *mgl*/*metC* and *porA*, which encode enzymes related to the above reactions, were also significantly up-regulated in this study. In order to test our hypothesis, we need to further detect the intracellular concentration of 2-ketobutyric acid in the future.

In conclusion, silkworm excrement after chlorophyll ethanol-extraction could be directly reused by *H. mediterranei* ATCC 33500 to accumulate PHBV without genetic manipulation of the stain. The success of open fermentation also reduced energy consumption to a certain extent, and the PHBV with high 3 HV content obtained by fermentation of silkworm excrement extract has higher product value. Therefore, we speculate that the PHA fermentation strategy developed in this study is suitable for biodegradable plastic applications with high 3 HV content demands.

## Data availability statement

The datasets presented in this study can be found in online repositories. The names of the repository/repositories and accession number(s) can be found in the article/Supplementary material.

## Author contributions

SC, YW, and LC: conceptualization and writing—original draft. SC, YW, RL, HJ, YQ, and MJ: data curation. SC, YW, YZ, and LC: funding acquisition. YZ, YM, and LC: investigation. XY, YZ, HJ, and SZ: methodology. LC: project administration. YZ and LC: supervision. All authors have read and agreed to the published version of the manuscript.

## Funding

This work was supported by grants from the National Natural Science Foundation of China (Grant no. 31700071), the Fundamental Special Funds for the Provincial Universities of Zhejiang (Grant no. JDK21005), and Quanzhou City Science & Technology Program of China (Grant no. 2019N113S).

## Conflict of interest

The authors declare that the research was conducted in the absence of any commercial or financial relationships that could be construed as a potential conflict of interest.

## Publisher’s note

All claims expressed in this article are solely those of the authors and do not necessarily represent those of their affiliated organizations, or those of the publisher, the editors and the reviewers. Any product that may be evaluated in this article, or claim that may be made by its manufacturer, is not guaranteed or endorsed by the publisher.

## Footnotes

^1^ http://blast.ncbi.nlm.nih.gov/Blast.cgi
